# Soluble ICAM-1, Independent of IL-6, Is Associated with Prevalent Frailty in Community-Dwelling Elderly Taiwanese People

**DOI:** 10.1371/journal.pone.0157877

**Published:** 2016-06-16

**Authors:** Wei-Ju Lee, Liang-Kung Chen, Chih-Kuang Liang, Li-Ning Peng, Shu-Ti Chiou, Pesus Chou

**Affiliations:** 1 Aging and Health Research Center, National Yang Ming University,Taipei City, Taiwan; 2 Institute of Public Health, National Yang Ming University, Taipei City, Taiwan; 3 Department of Family Medicine, Taipei Veterans General Hospital Yuanshan Branch,Yilan County, Taiwan; 4 Center for Geriatrics and Gerontology, Taipei Veterans General Hospital, Taipei City, Taiwan; 5 Center for Geriatrics and Gerontology, Kaohsiung Veterans General Hospital, Kaoshiung City, Taiwan; 6 Health Promotion Administration, Ministry of Health and Welfare,Taipei City Taiwan; University of Padova, ITALY

## Abstract

**Background:**

Activation of inflammatory pathway with elevation of inflammatory biomarkers such as Interleukin 6 (IL-6) has been considered a pathophysiological feature of frailty. In recent years, the association between Intercellular adhesive molecule -1 (ICAM-1) and vascular inflammatory was established. Provocation of inflammatory cascades from ICAM-1 is potential IL-6 related, although the association between the inflammatory process and frailty is little to known. The study was intended to evaluate the relationship between serum ICAM-1, IL-6 and frailty.

**Materials and Methods:**

Data was derived from a representative national sampling cohort in Taiwan. The cross-sectional study included nine-hundred-forty-six community-dwelling people aged 53 and older. Frailty was defined as having three or more components (including, muscle shrinkage, slowness, weakness, exhaustion, and low activity) Serum IL-6 and ICAM-1 levels were measured using standard enzyme–linked immunosorbent assays.

**Results:**

Soluble ICAM-1 (sICAM-1) levels were stepwise increased in non-frail, pre-frail and frail elderly people (the median levels were 255 vs. 265 vs. 285 ng/ml, respectively p<0.001). A multivariate multinomial logistic regression, which was adjusted for age, sex, smoking, education, BMI, and chronic disease number, was utilized to determine that the probability of being frail due to increased log (ICAM-1) and log (IL-6) standard deviation levels were 1.44 (95% CI 1.09–1.91) and 1.54 (95%CI 1.07–2.20), respectively.

**Conclusion:**

sICAM-1 was significantly associated with frailty, independent of IL-6. This implied that leukocyte migration and inflammation cascade activation might contribute to frailty, in addition to monocyte/macrophage-mediated immuno-inflammation.

## Introduction

Frailty, which is one of the most important advances in geriatrics over the last ten years, has been recognized as a geriatric syndrome resulting from multisystem dysregulation, and it presents with a decreasing physical reservoir and increasing vulnerability to stress [1]. The estimated frailty prevalence, which varies according to its definitions, ranges from 7% to 28% [1, 2]. Frail older adults have a higher risk for adverse health outcomes, including falling, hospitalization, and mortality [1, 3]. Several longitudinal studies have suggested that one of the most important pathways of frailty development is the immune/inflammatory pathway [4–7].

The InCHIANTI study showed a significant negative association between interleukin-6 (IL-6) levels and physical performance [4]. The Women’s Health and Aging Study(WHAS) found that heightened inflammatory states were manifested by elevated levels of inflammatory biomarkers, such as IL-6 and white blood cells, in frail elderly people [5]. The Health ABC study found that higher IL-6 and tumor necrosis factor *α* (TNF-*α*) cytokine levels were associated with a loss of muscle mass and strength in healthy older adults, which are two central components of frailty [6]. An *in vitro* study revealed that peripheral monocytes stimulated with lipopolysaccharides produced more IL-6 in frail adults compared with non-frail adults [8]. These cross-sectional studies imply that IL-6 may play a central role in the emerging frailty pathway. However, a longitudinal study of 1,720 community-dwelling elderly people with a 3-year follow-up failed to demonstrate any relationship between IL-6 and frailty prevalence or incidence [9].

Intercellular adhesion molecule (ICAM)-1, an immunoglobulin-like cell adhesion molecule, is expressed by several cell types, including leukocytes and endothelial cells. ICAM-1 not only acts as a leukocyte adhesion molecule to facilitate transmigration of leukocytes out of vessels and into tissues but also contributes to inflammatory responses [10]. The soluble or circulating form of ICAM-1 (sICAM-1) is composed of the extracellular region of ICAM-1 and reflects ICAM-1 expression in endothelial cells [11]. Studies suggest that changes in sICAM-1 levels are associated with atherosclerosis, cerebral small vessels disease [12], autoimmune disorders [13,14], various cancers [15], and mortality [16]. *In vitro* studies showed that sICAM-1 enhanced the production of IL-6,TNF- *α*, and macrophage inflammatory protein-2 (MIP-2) after incubating with external stimuli, which suggests that sICAM-1 activates pro-inflammatory cascades [17,18].

Concluded these evidences, ICAM-1 was associated with vascular inflammatory and may activate pro-inflammatory pathway other than IL-6 specific ones. Thus, the study was intended to investigate the association of sICAM-1 and frailty. We hypothesized that elevated sICAM-1 would be significantly associated with frailty, independent of IL-6. Here, we provide initial evidence for this hypothesis by relating endothelial cell activation to frailty.

## Materials and Methods

### Study subjects

**Fig 1:**
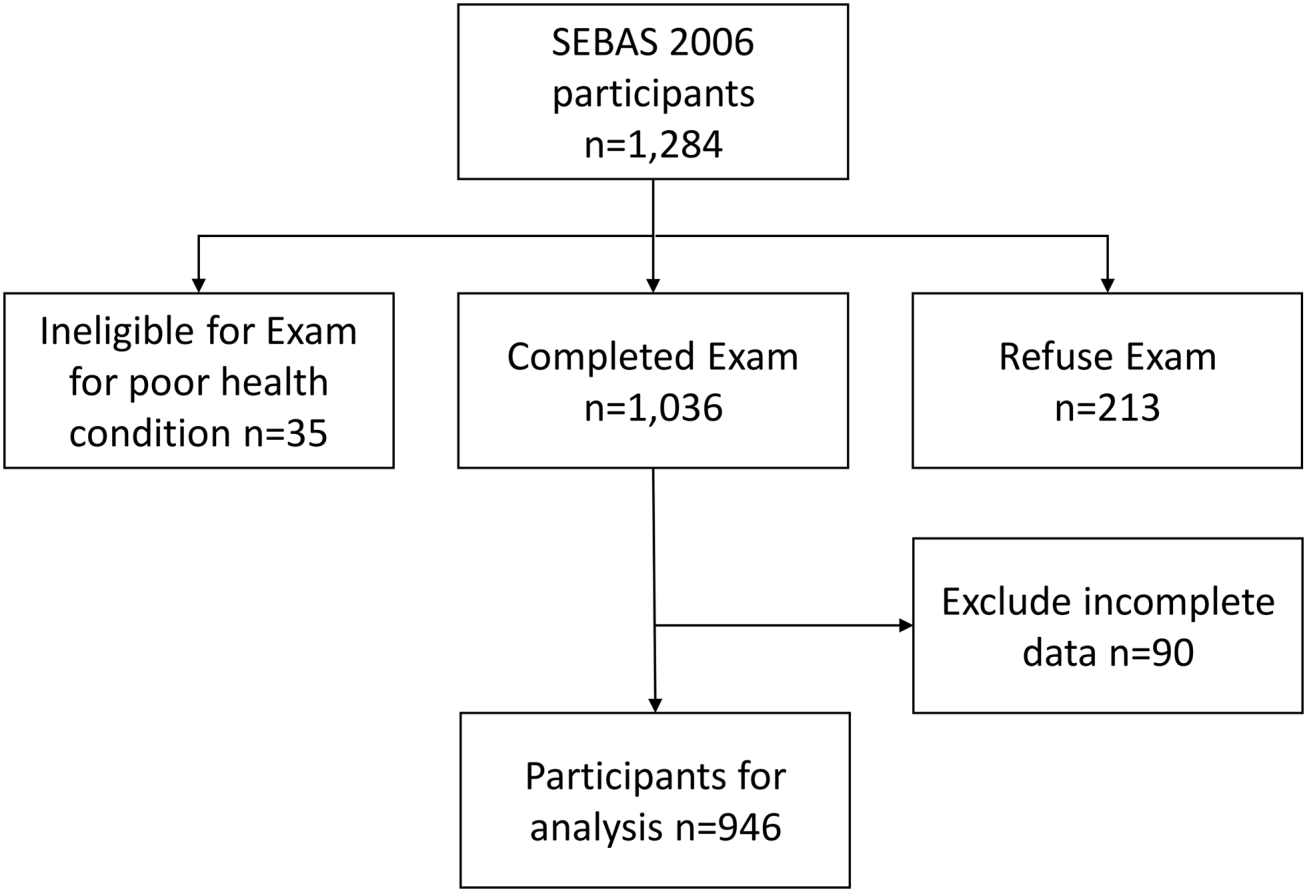
Participants derived from the Social Environment and Biomarkers of Aging Study 2006 for analysis.

The Social Environment and Biomarkers of Aging Study (SEBAS), a national representative population-based cohort study, was intended to examine the antecedents, correlates, and consequences of changes in biological measures and health. Participants were drawn according to a multistage and proportional sampling strategy, which included Taiwanese subjects that were 53 years of age and older. The details of the research design, sampling strategy, and cohort profile have been reported elsewhere [19]. The data included in this study were from the second SEBAS wave in 2006. Overall, 1,034 of 1,284 respondents received complete physical examinations, including muscle strength, gait speed, and laboratory examinations. The missing and incomplete data from 88 respondents were excluded from the analysis. The data of the remaining 946 respondents were used for the analysis (Fig 1).

Princeton University, Georgetown University, and the Joint Institutional Review Boards of Taiwan approved the entire study, and written informed consents were obtained from all of the participants.

### Frailty definition

Based on the frailty definition from the Cardiovascular Health Study, five physical components were selected to describe a frailty phenotype [1]. Among the five components, shrinkage was defined when the albumin serum levels of the study subjects were <3.3 g/dl [20]. Exhaustion was evaluated based on two questions from a modified 10-item Center for Epidemiological Studies-Depression scale (CES-D) in the Fried’s criteria [21], which included, “In the past week, have you experienced feelings of exhaustion?” and “In the past week, have you experienced that you were unable to gather your energy to do things?” If the answer was yes, then we asked how often this happened. Participants that answered sometimes (2–3 days per week) or often (more than 4 days a week) for the either one of abovementioned questions were classified as exhausted. In the SEBAS, all of the participants were asked whether they engaged in regular physical activities or leisure physical activities, such as Chi Kong and Tai Chai. Physical activity was measured by a weighted score that was summated from the type and frequency of exercise and leisure activities [22]. A consensus was held to determine the weighted scoring system [23] Briefly, Men with scores less than 3 and women with scores less than 2 were considered to be physically inactive in this study. Walking speed was measured with a 3-meter walking evaluation. Slowness was defined as the lowest quintile of the walking speed for all of the participants. Additionally, handgrip strength was measured with a North CoastTM hydraulic hand dynamometer (NC70142) to evaluate weakness. The best measurement from the three trials of participants’ dominant hand was recorded. Weakness was defined as the lowest handgrip strength quintile of all of the participants. Those with three or more phenotypes were defined as frailty, and those with one or two phenotypes were defined as prefrail.

### Biological markers

All of the participants fasted overnight and had venous blood samples drawn by a research nurse. The blood samples were immediately shipped from the hospital to Union Clinical Laboratory (UCL) in Taipei by noon and were processed according to the standard laboratory protocol. The serum IL-6 and ICAM-1 levels were measured using commercially available sandwiched-type enzyme-linked immunosorbent assay kits (ELISAs, Quantikine; R&D Systems). The IL-6 and ICAM-1 immunoassays had sensitivities of 0.7 pg/ml and 0.35 ng/ml and inter-assay coefficient of variance percentages of 12.1% and 16.9%, respectively [19].

### Healthy behavior and chronic disease

In this study, tobacco smoking status was defined as a current smoker, smoking in the past six months, ex-smoker (someone who quit smoking), and never smoked. There were fourteen self-reported physician-diagnosed common chronic diseases collected in the SEBAS, including hypertension, diabetes, heart disease, stroke, cancer, pulmonary disease, arthritis, liver disease, kidney disease, hip fracture, cataract, gout, and osteoarthritis. The chronic disease numbers were evaluated.

### Statistical analysis

In this study, continuous variables were expressed as the means ± standard deviation and medians ± interquartile range (for variables that were not normally distributed, tested by the normal probability plot). The categorical data were expressed as frequencies and percentages. Continuous variable comparisons were performed with one-way ANOVAs. The categorical data were compared with the Chi-square test, Fisher’s exact test, or a crosstabs analysis when appropriate. Log transformation of the IL-6 and ICAM-1 values was adopted to avoid a potentially skewed distribution of the biomarkers. Frailty was categorized into three levels (non-frail vs. pre-frail vs. frail). Multicolinearity were tested between variables, and VIF (variance inflation factor) was used to quantify the severity of collinearity. A multinomial logistic regression was applied to explore the association between the serum IL-6 levels, serum ICAM-1 levels, and frailty. First, we used continuous log IL-6 and log ICAM-1 covariates to obtain maximal statistical efficiency. Because the biomarkers of interest have different units, the results of the logistic regressions were not easily comparable. We standardized the biomarker distributions and expressed the estimated effects as the probability of being frail vs. non-frail or pre-frail vs. non-frail for every unit increase in the log IL-6 and log ICAM-1 standard deviation. To explore possible non-linear relationships, each biomarker was categorized into tertiles for analysis. A p-value (2-tailed) less than 0.05 was considered statistically significant. All of the analyses were performed with the SAS statistical package, version 9.4 (SAS Institute, Inc., Cary, NC) and SPSS for Windows version 20.0 (IBM, Inc., Chicago, IL).

## Results

Nine-hundred-forty-six participants with complete demographic data and serum IL-6/ICAM-1 data were included for analysis. The demographic characteristics are shown in Table 1. The subject mean age was 65.5 ± 9.4 years, and the mean chronic disease number was 1.6. There were 85 (9.0%) frail, 531 (56.1%) pre-frail and 330 (34.9%) non-frail adults. Significant differences in age, gender, education, tobacco, alcohol use, and chronic disease numbers were observed, whereas the body mass index (BMI) differences were insignificant.

**Table 1:** Demographic characteristics and serum ICAM-1 and IL-6 levels among the participants according to their frailty status.

Variable	Total	Non-frail	Pre-frail	Frail	*p*
Number	946	330	531	85	
ICAM-1 (ng/ml)					
mean±SD	276.4±96.2	263.3±88.9	276.7±91.5	325.3±131.2	<0.001
Median (IQR)	265.0(225.0–310.0)	255.0(210.0–300.0)	265.0(225.0–315.0)	285.0(250.0–360.0)	
IL-6 levels (pg/ml)					<0.001
mean±SD	4.0±6.2	3.8±7.2	3.9±5.1	5.7±8.2	
Median (IQR)	2.5(2.0–4.0)	2.5(1.5–3.5)	2.5(2.0–4.0)	4.0(2.5–5.5)	
Age, mean±SD (year)	65.5±9.4	63.2±8.2	65.5±9.4	74.4±7.9	<0.001
Sex, Men,rate (%)	520(100%)	51.8	40.5	47.1	0.005
Education, mean±SD (year)	7.4±4.9	8.7±5.0	7.2±4.6	4.1±4.4	<0.001
BMI, mean±SD (kg/m^2^)	24.8±3.5	24.8±3.4	24.9±3.4	24.6±4.0	0.737
Smoke, rate (%)					<0.001
Never	583(100%)	40.0	51.5	8.6	
Former	180(100%)	31.1	57.2	11.7	
Current	183(100%)	22.4	70.0	7.7	
Number of chronic diseases, median(IQR)	1.0(0.0–2.0)	1.0(0.0–2.0)	1.0(0.0–2.0)	3.0(1.0–4.0)	<0.001

The log ICAM-1 and log IL-6 mean and median values increased stepwise across the frailty categories. The frailty percentages increased from the bottom to the top tertile for both ICAM-1 and IL-6. VIF of all variable were less than 2.5. In multinomial analysis, continuous log ICAM-1 and log IL-6 measurements were significantly associated with frailty, as shown in Table 2, after adjusting for age, gender, education, smoking, BMI, and chronic disease number. The OR of being frail for log ICAM-1 and log IL-6 were 1.44 and 1.54, respectively. Compared with the bottom ICAM-1 tertile, the OR of being frail in the middle and top tertiles was 2.57 and 2.43, respectively. Compared with the bottom IL-6 tertile, the OR of being frail was only significant in the top tertile (OR 2.34, 95% CI 1.14–4.76). When adding IL-6 for adjustment, log ICAM-1 remained significant associated with frailty (OR 1.36, 95% CI 1.03–1.82). (Model III)

**Table 2:** Multinomial logistic regression analysis to explore the association among the log ICAM-1 values, log IL-6 values, and frailty status using continuous measurements and serum ICAM-1 and I-6 level tertile categories.

	Model I		Model II		Model III	
	Pre-frail	Frail	Pre-frail	Frail	Pre-frail	Frail
Using continuous measurements						
Log (ICAM-1)						
OR(95%CI)	1.06(0.91–1.23)	1.44(1.09–1.91)			1.05(0.90–1.22)	1.36(1.03–1.82)
Log (IL-6)						
OR(95%CI)			1.08(0.94–1.25)	1.54(1.07–2.20)	1.08(0.93–1.25)	1.41(0.98–2.03)
Using tertiles						
ICAM-1 tertiles						
Bottom	reference	reference			reference	reference
Middle						
OR(95%CI)	1.03(0.73–1.46)	2.57(1.14–5.80)			1.03(0.73–1.45)	2.57(1.13–5.82)
Top						
OR(95%CI)	1.10(0.76–1.59)	2.43(1.08–5.44)			1.08(0.74–1.57)	2.21(0.97–4.98)
IL-6 tertiles						
Bottom			reference	reference	reference	reference
Middle						
OR(95%CI)			1.07(0.76–1.50)	1.28(0.60–2.74)	1.06(0.76–1.49)	1.26(0.58–2.71)
Top						
OR(95%CI)			1.16(0.80–1.69)	2.34(1.14–4.76)	1.15(0.79–1.67)	2.28(1.10–4.89)

Adjusted for age, gender, education, smoke, chronic disease number, and BMI;

## Discussion

In this study, we observed an association between sICAM-1 and frailty for the first time after adjusting for IL-6, age, gender, BMI, education, and chronic disease number.

It is well known that sICAM-1 plays a role in leukocyte emigration. The physiological process of sICAM-1 is mediated through outside-in signaling, which (1) re-constructs the actin cytoskeleton to facilitate leukocyte migration and (2) activates pro-inflammatory cascades [10]. The sICAM-1 mean and standard deviation levels in our study were 276±96 ng/ml, which is consistent with previous Danish and U.S. study findings [24,25]. The elevation in the sICAM-1 levels in frail individuals in this study was not as marked as for levels observed in tumors, such as in lung cancer (>414 ng/ml) [26] and breast cancer, as well as with HIV infection (1,086 ng/ml) [27]; however, they were similar to values for the prediction of future myocardial infraction risk (>260 ng/ml) [28] and severe sepsis (>444 ng/ml) [29].

The higher sICAM-1 levels in frail elderly adults that were observed in this study suggest that leukocyte migration and the activation of inflammatory processes contribute to frailty. sICAM-1 was elevated in protein energy wasting syndrome, which was highly associated with frailty in chronic kidney diseases [30].

Many studies found that high IL-6 levels were associated with the presentation of physical frailty [4,5,31]. In our study, IL-6 was significantly associated with frailty (Table 2 model II), which was consistent with previous studies. This implied that frailty is associated with monocyte and macrophage activation. A significant association between sICAM-1 and IL-6 was observed in this study, which might indicate the basic physiological functions of leukocyte migration and pro-inflammatory cascade activation, including IL-6. Both sICAM-1 and IL-6 were not significant associated with prefrail, which was similar with previously report.[5] The results from our study showed a significant association between sICAM-1 and frailty after adjusting for IL-6 (Table 2, Model III). This suggested that the activation of inflammatory processes through sICAM-1 contributes, at least partially, to frailty.

Vicious cycle of frailty proposed by Fried suggested that frailty developed from sarcopenia resulted from inflammatory.[1] A U-shape association between frailty and BMI was reported which denoted that both extremely low and high BMI were associated with frailty. [32] It was supposed to explain why BMI did not reach statistical significance in the study.

There were two important implications of our findings. First, it provided a basis for further investments to investigate the mechanisms and immunopathophysiology of frailty. Second, sICAM-1 might be an attractive therapeutic target to block leukocyte/endothelium interactions and ongoing inflammatory responses [33,34], which might enable its application to frailty intervention.

Despite all efforts ran into the study, there were still several limitations. First, the modified frailty definition with unintentional weight loss and low activity was adopted, which could have influenced the frailty prevalence. Although the frailty percentage (9%) in the study was similar to a CHS cohort (7%) [1] and another Taiwanese cohort (5%) [23], the modification of CHS frailty might introduce a misclassification bias. Second, this study was limited to a cross-sectional study design; therefore, the causal relationship between sICAM-1 and frailty could not be established in the current study.

In conclusion, sICAM-1 and IL-6 were stepwise increased in the non-frail, pre-frail, and frail categories. The multivariate multinomial logistic analysis showed that sICAM-1 was significantly associated with frailty and was independent of IL-6. This implies that leukocyte migration and inflammation cascade activation might contribute to frailty, in addition to monocyte/macrophage-mediated immuno-inflammation.
